# In Vitro and In Vivo Antifungal Activity of Sorbicillinoids Produced by *Trichoderma longibrachiatum*

**DOI:** 10.3390/jof7060428

**Published:** 2021-05-28

**Authors:** Men Thi Ngo, Minh Van Nguyen, Jae Woo Han, Myung Soo Park, Hun Kim, Gyung Ja Choi

**Affiliations:** 1 Center for Eco-Friendly New Materials, Korea Research Institute of Chemical Technology, Daejeon 34114, Korea; ngomen@krict.re.kr (M.T.N.); nvminh@krict.re.kr (M.V.N.); jaewoo82@krict.re.kr (J.W.H.); 2 Department of Medicinal Chemistry and Pharmacology, University Science and Technology, Daejeon 34113, Korea; 3 School of Biological Sciences, Seoul National University, Seoul 08826, Korea; ms1014@snu.ac.kr

**Keywords:** *Trichoderma longibrachiatum*, sorbicillinoid, antifungal compound, plant pathogen

## Abstract

In the search for antifungal agents from marine resources, we recently found that the culture filtrate of *Trichoderma longibrachiatum* SFC100166 effectively suppressed the development of tomato gray mold, rice blast, and tomato late blight. The culture filtrate was then successively extracted with ethyl acetate and n-butanol to identify the fungicidal metabolites. Consequently, a new compound, spirosorbicillinol D (**1**), and a new natural compound, 2′,3′-dihydro-epoxysorbicillinol (**2**), together with 11 known compounds (**3**–**13**), were obtained from the solvent extracts. The chemical structures were determined by spectroscopic analyses and comparison with literature values. The results of the in vitro antifungal assay showed that of the tested fungal pathogens, *Phytophthora infestans* was the fungus most sensitive to the isolated compounds, with MIC values ranging from 6.3 to 400 µg/mL, except for trichotetronine (**9**) and trichodimerol (**10**). When tomato plants were treated with the representative compounds (**4**, **6**, **7**, and **11**), bisvertinolone (**6**) strongly reduced the development of tomato late blight disease compared to the untreated control. Taken together, our results revealed that the culture filtrate of *T. longibrachiatum* SFC100166 and its metabolites could be useful sources for the development of new natural agents to control late blight caused by *P. infestans*.

## 1. Introduction

Phytopathogenic fungi have consistently threatened crops, causing serious damage that results in the loss of crop yields and reduces the quality of crops worldwide [1,2]. In an effort to protect plants, chemical fungicides have been mainly used, but the overuse of these fungicides has also led to side effects, such as environmental pollution, toxicity to humans and animals, and resistance of fungal pathogens [3]. Therefore, the development of new fungicides that are safer and more eco-friendly has been emphasized, and biofungicides using plant extracts or useful microbes have been considered as an alternative that can replace synthetic fungicides [4,5,6]. More recently, the integrational use of biofungicides with conventional fungicides has been highlighted to reduce the chemical fungicide use and to avoid fungicide resistance [7,8].

To date, there are over 23,000 known microbial secondary metabolites, which are significant sources for life-saving drugs, and 42% of these known microbial secondary metabolites are produced by fungi [9,10]. Many studies have reported on the potential efficacy of fungi or fungal metabolites in controlling plant diseases caused by fungal pathogens. Of the many fungal species, *Trichoderma* spp. have been widely studied and used in the biological control against phytopathogenic fungi because of their high capacity to secrete large amounts of enzymes and metabolites [11]. In particular, *Trichoderma harzianum* and *Trichoderma viride* have been developed as commercial products, which are now used to control foliar and soil-borne diseases in various crops [12]. In addition to their antifungal activity, it has been reported that *Trichoderma* spp. promote plant growth and productivity by inducing systemic resistance and enhancing nutrient efficiency [13].

The fungus *T. longibrachiatum* is generally isolated from terrestrial soil and plants and has broad-spectrum and potential antagonistic effects in inhibiting the growth of plant pathogenic fungi [14,15,16,17]. In terms of the antifungal compounds derived from *T. longibrachiatum*, it has been reported that a new rare norsesquiterpene exhibited a potent antagonistic ability against *Colletotrichum lagenarium*, *C. fragariae*, carbendazim-resistant strains of *Botrytis cinerea*, and *Fusarium* spp. [15]. However, there is still a lack of papers describing the in vitro and in vivo antifungal activities of secondary metabolites produced by *T. longibrachiatum* against plant diseases caused by fungi.

The current study focused on the identification of antifungal metabolites produced by *T. longibrachiatum* SFC100166 and the evaluation of their ability to control plant diseases in vivo. To this end, we isolated active compounds from the culture filtrate of *T. longibrachiatum* SFC100166 based on bioassay-guided fractionation, which resulted in the identification of 13 compounds. Here, we report the isolation and structural elucidation of these compounds and their in vitro and in vivo antifungal activities against plant pathogenic fungi.

## 2. Materials and Methods

### 2.1. Fungal Strains and Culture Conditions

The fungus SFC100166 was isolated from the foreshore soil at Ganghwa-gun, Incheon, Korea, and deposited as a patent microorganism to the Korean Agricultural Culture Collection (KACC, Jeonju, Korea; No. KACC 83038BP). The fungus was maintained on potato dextrose agar (PDA) medium at 25 °C. Twenty agar plugs punched by a 6 mm-diameter cork-borer from SFC100166 culture plates were inoculated into 400 mL of potato dextrose broth (PDB) and then incubated at 25 °C for 10 days to isolate the antifungal compounds from the SFC100166 culture.

For the in vitro antifungal activity assay, seven phytopathogenic fungi provided by the KACC were used: *Alternaria brassicicola* (KACC 40036), *B. cinerea* (KACC 48736), *Colletotrichum coccodes* (KACC 48737), *Cladosporium cucumerinum* (KACC 40576), *Cylindrocarpon destructans* (KACC 41077), *Magnaporthe oryzae* (KACC 46552), and *Phytophthora infestans* (KACC 48738). Additionally, we used two obligate parasitic fungi, *Puccinia triticina* and *Blumeria graminis* f. sp. *Hordei*, for the in vivo antifungal activity, which were maintained on their host plants. Maintenance and preparation of the plant pathogens for the in vitro and in vivo antifungal assay were performed as previously described [18,19].

### 2.2. Genomic DNA Extraction and Phylogenetic Analysis

For the isolation of genomic DNA (gDNA), the SFC100166 isolate was grown in 50 mL of PDB medium at 25 °C for 4 days on a rotary shaker (150 rpm), and the gDNA was extracted using the cetyltrimethylammonium bromide (CTAB) procedure as previously described [20]. For phylogenetic analysis, the translation elongation factor 1-*α* (*TEF1*) gene was amplified by primer sets Tef1-F (5′-GGTACTGGTGAGTTCGAGGCTG-3′) and Tef1-R (5′-GGGCTCGATGGAGTCGATAG-3′). The resulting amplicon was purified using the GeneAll ExpinTM PCR purification kit (GeneAll, Seoul, Korea) and then analyzed by a sequencing analysis service (Macrogen, Daejeon, Korea). The resulting sequence was analyzed with the BLASTn program of the NCBI (http://www.ncbi.nlm.nih.gov). The sequences were aligned using ClustalW implemented in MEGA version 7, and distances were estimated based on the model [21]. A phylogenetic tree was generated using the neighbor-joining method with 1000 bootstrap analyses [22].

### 2.3. Isolation of Antifungal Compounds

An isolation scheme of active compounds from the fungus *T. longibrachiatum* SFC100166 is shown in Supplemental Figure S1. A ten-day-old fungal culture (10 L) of *T. longibrachiatum* SFC100166 was filtered with two layers of filter paper circles and then partitioned with an equal volume of ethyl acetate (EtOAc) and n-butanol (BuOH), sequentially. Each layer was concentrated to dryness to give a residue of EtOAc extract (2.6 g), BuOH extract (1.8 g), and water-soluble fraction (16.7 g).

The EtOAc extract was separated by a medium pressure liquid chromatography (MPLC) system (Isolera One; Biotage, Uppsala, Sweden) equipped with a SNAP KP-Sil 50 g cartridge (Biotage), eluting with a gradient mixture of methylene chloride/EtOAc (85:15 to 0:100, *v*/*v*) to give seven fractions, E1–E7. Based on in vitro antifungal activity-guided fractionation, the active fraction E2 (118.7 mg) was further purified by an LC-20AR HPLC system (Shimadzu, Kyoto, Japan) equipped with a Capcell Pak C18 column (20 × 250 mm i.d., 5 μm; Shiseido Co., Ltd., Tokyo, Japan) using an isocratic elution of 50% aqueous acetonitrile (ACN) containing 0.1% formic acid to yield two pure compounds, **7** (45.4 mg) and **8** (6.8 mg). Another active fraction, E3 (315.5 mg), was subjected to Sephadex LH-20 gel column chromatography (3 × 100 cm i.d., 25–100 μm; Amersham Biosciences, Sweden) using an isocratic elution of methanol (MeOH) to obtain compound **12** (16.1 mg) and the active fraction E31 (218.1 mg). Fraction E31 was continuously chromatographed by prep HPLC and eluted with an isocratic mixture of 79% aqueous MeOH containing 0.1% formic acid, giving pure compounds **6** (124.2 mg) and **10** (14.2 mg). E5 (294.6 mg) was also an active fraction, purified by prep HPLC with an isocratic mixture of 60% aqueous MeOH to yield compounds **4** (45.7 mg) and **5** (73.7 mg). Similar to the separation of E3, fraction E6 (273.9 mg) was also applied to the Sephadex LH-20 gel column chromatography (3 × 100 cm i.d.) with solvent elution of MeOH to give two pure compounds, **9** (64.9 mg) and **13** (11.9 mg), and a remaining active fraction, E63 (60.6 mg), was further purified by prep HPLC, eluting with an isocratic mixture of 65% aqueous MeOH to yield compounds **1** (17.0 mg) and **3** (28.2 mg).

The BuOH extract was loaded onto a SNAP Ultra C18 60 g cartridge (Biotage) in an MPLC system using a stepwise gradient elution of aqueous MeOH containing 0.1% formic acid (20 to 100%, *v*/*v*), giving six factions, B1–B6. Based on the in vitro antifungal activity-guided fractionation, the active fraction B2 (355.0 mg) was recrystallized to obtain compound **11** (289.0 mg), and B3 (78.4 mg) was further separated by prep HPLC eluting with an isocratic mixture of 35% aqueous ACN containing 0.1% formic acid to yield pure compound **2** (7.6 mg).

### 2.4. Spectral Methods

The chemical structures of the purified compounds were determined by spectroscopic analyses and comparisons with values in previous literature. High-resolution electrospray ionization mass spectrometry (HRESIMS) data were determined by a Synapt G2 system (Waters Co., Milford, MA, USA). The 1D and 2D nuclear magnetic resonance (NMR) spectra were recorded by a Bruker Advance 400 MHz spectrometer (Burker BioSpin, Rheinstetten, Germany) at 400 MHz for ^1^H and 100 MHz for ^13^C in MeOH-*d*_4_ (Cambridge Isotope Laboratories, Tewksbury, MA, USA). Chemical shifts were referenced to the solvent peaks (*δ*_H_ 4.87 and *δ*_C_ 49.0 for MeOH-*d*_4_).

### 2.5. Spectral Data

#### 2.5.1. Spirosorbicillinol D (**1**)

Yellowish amorphous powder; ${\left[\alpha \right]}_{D}^{25}$ + 142 (*c* 0.05, MeOH); UV (MeOH) *λ*max (log *ε*) 208 (4.2), 364 (4.3); ^1^H and ^13^C NMR spectroscopic data, see Table 1; high-resolution electrospray ionization mass spectrometry (HRESIMS) *m/z* 511.1580 [M + Na]^+^ (calculated for C_25_H_28_O_10_Na, 511.1580).

#### 2.5.2. 2′,3′-Dihydro-epoxysorbicillinol (**2**)

Viscous yellow oil; UV (MeOH) *λ*max (log *ε*) 277 (4.3); ^1^H NMR (400 MHz, MeOD): ***δ***_H_ 5.48 (2H, p, *J* = 6.0 Hz), 2.67 (2H, m), 2.28 (2H, m), 1.69 (3H, s), 1.65 (3H, d, *J* = 5.4 Hz), 1.48 (3H, s); ESI-MS: *m/z* 289 [M + Na]^+^ and 265 [M − H]^−^.

#### 2.5.3. Spirosorbicillinol A (**3**)

Yellowish amorphous powder; ${\left[\alpha \right]}_{D}^{25}$ + 184 (*c* 0.05, MeOH); UV (MeOH) *λ*max (log *ε*) 208 (4.1), 364 (4.3); ^13^C NMR spectroscopic data, see Table 1; ESI-MS: *m/z* 511 [M + Na]^+^.

#### 2.5.4. Spirosorbicillinol B (**4**)

Yellowish amorphous powder; ${\left[\alpha \right]}_{D}^{25}$ + 428 (*c* 0.05, MeOH); UV (MeOH) *λ*max (log *ε*) 208 (4.3), 364 (4.5); ^13^C NMR spectroscopic data, see Table 1; ESI-MS: *m/z* 511 [M + Na]^+^.

#### 2.5.5. Spirosorbicillinol C (**5**)

Yellowish amorphous powder; ${\left[\alpha \right]}_{D}^{25}$ + 464 (*c* 0.05, MeOH); UV (MeOH) *λ*max (log *ε*) 208 (4.1), 363 (4.2); ^13^C NMR spectroscopic data, see Table 1; ESI-MS: *m/z* 511 [M + Na]^+^.

#### 2.5.6. Bisvertinolone (**6**)

Yellowish amorphous powder; UV (MeOH) *λ*max (log *ε*) 240 (4.3), 275 (4.5), 394 (4.2); ^1^H and ^13^C NMR spectroscopic data, see Table S1; ESI-MS *m/z* 535 [M + Na]^+^.

#### 2.5.7. Bisorbicillinol (**7**)

Yellowish amorphous powder; UV (MeOH) *λ*max (log *ε*) 285 (4.5), 300 (4.5), 390 (4.1); ^13^C NMR spectroscopic data, see Table S1; ESI-MS *m/z* 519 [M + Na]^+^ and 495 [M − H]^−^.

#### 2.5.8. Bisvertinoquinol (**8**)

Yellow powder; UV (MeOH) *λ*max (log *ε*) 250 (3.9), 301 (4.3), 370 (4.1), 380 (4.1), 400 (3.9); ^13^C NMR spectroscopic data, see Table S1; ESI-MS *m/z* 521 [M + Na]^+^ and 497 [M − H]^−^.

#### 2.5.9. Trichotetronine (**9**)

Yellowish amorphous powder; UV (MeOH) *λ*max (log *ε*) 271 (4.6), 291 (4.6), 384 (4.3); ^13^C NMR spectroscopic data, see Table S1; ESI-MS *m/z* 519 [M + Na]^+^ and 495 [M − H]^−^.

#### 2.5.10. Trichodimerol (**10**)

Pale yellow powder; UV (MeOH) *λ*max (log *ε*) 240 (4.0), 295 (4.2), 307 (4.2), 362 (4.5); ^13^C NMR spectroscopic data, see Table S1; ESI-MS *m/z* 519 [M + Na]^+^ and 495 [M − H]^−^.

#### 2.5.11. Epoxysorbicillinol (**11**)

Yellowish amorphous powder; UV (MeOH) *λ*max (log *ε*) 287 (4.2); ^13^C NMR spectroscopic data, see Table S1; ESI-MS *m/z* 287 [M + Na]^+^.

#### 2.5.12. Oxosorbicillinol (**12**)

Yellowish amorphous powder; UV (MeOH) *λ*max (log *ε*) 231 (4.0), 301 (3.9), 378 (4.2); ^13^C NMR spectroscopic data, see Table S1; ESI-MS *m/z* 287 [M + Na]^+^ and 263 [M − H]^−^.

#### 2.5.13. Cyclonerodiol (**13**)

Colorless oil; UV (MeOH) no UV above 210 nm; ^13^C NMR spectroscopic data, see Table S1.

### 2.6. Microdilution Broth Assay for In Vitro Antimicrobial Activity

Minimum inhibitory concentration (MIC) values of the purified compounds against phytopathogenic fungi were determined by the broth microdilution assay using the 2-fold serial dilution method as described by the modified CLSI M38-A method [23]. Briefly, a spore suspension (1 × 10^5^ spores/mL of PDB) of each fungal pathogen was added to the wells of a 96-well microtiter plate, and then the stock solutions of the compounds dissolved in DMSO were added at an initial concentration of 400 μg/mL. The microtiter plates were incubated for 1–3 days, and the MIC values were determined by visual inspection of complete growth inhibition. The chemical blasticidin-S and a PDB medium containing 1% DMSO were used as a positive and negative control, respectively. The assay was performed two times with three replicates for each compound at all concentrations investigated.

### 2.7. Evaluation of Fungal Disease Control Efficacy

To investigate the disease control efficacy of the culture filtrate, solvent extracts, and pure compounds, we used six kinds of fungal plant diseases: rice blast (RCB; caused by *M. oryzae*), tomato late blight (TLB; caused by *P. infestans*), tomato gray mold (TGM; caused by *B. cinerea*), wheat leaf rust (WLR; caused by *P. triticinia*)*,* barley powdery mildew (BPM; caused by *B. graminis* f. sp. *hordei*), and pepper anthracnose (PAN; caused by *C. coccodes*). The culture filtrate containing 0.025% Tween 20 solution was directly applied onto the plant, and the extracts (1000 µg/mL) and pure compounds (125, 250, and 500 µg/mL) were prepared by dissolving in 5% MeOH solution containing 0.025% Tween 20 solution. Chemical fungicides (blasticidin-S, fludioxonil, dimethomorph, flusilazole, benomyl, and dithianon) were used as a positive control, whereas a 5% MeOH solution was used as a negative control. As host plants for the pathogens, we used rice (*Oryza sativa* L, cv. Chucheong), tomato (*Solanum lycopersicum* cv. Seokwang), wheat (*Triticum aestivum* cv. Geumgang), barley (*Hordeum sativum* cv. Hanyoung), and pepper (*Capsicum annuum* cv. Hyangchon), which were grown in a greenhouse at 25 ± 5 °C for 3–4 weeks. After treatment with the solvent extracts or pure compounds, the plants were inoculated with a fungal pathogen and incubated as previously described [4,5]. Disease severity based on the lesion area of the leaves was evaluated 3–7 days after inoculation, and the disease control efficacy was calculated with the following equation: control efficacy (%) = 100 × (1 − B/A), where A is the mean of the lesion area (%) on the leaves of the control plants and B is the mean of lesion area (%) on the leaves of the treated plants [6]. All experiments were conducted twice with three replicates for each treatment.

## 3. Results and Discussion

### 3.1. Disease Suppression by the Culture Filtrate and Its Extracts of the SFC100166 Isolate

During the course of screening for a culture filtrate with a potent efficacy in controlling plant diseases caused by fungi, we found that a culture filtrate of the SFC100166 isolate exhibited an inhibitory effect on the development of RCB, TGM, and TLB with control values of 85%, 94%, and 90%, respectively (Table 2). To identify the active compounds produced by the SFC100166 isolate, the culture filtrate was successively partitioned by the organic solvents EtOAc and BuOH, and then the yielding extracts were investigated for their ability to control six plant diseases caused by fungal pathogens. Both the EtOAc and BuOH extracts caused a reduction in the development of TGM and TLB over at least the control values of 80% (Table 2). In particular, the EtOAc extract showed a broad range of disease control efficacy on RCB, WLR, PAN, TGM, and TLB, with control values of over 60%. The suppression of BPM by the organic extracts was not found, and the remaining water extract did not show any suppressive effects on disease development caused by fungi. Therefore, we used the EtOAc and BuOH extracts for the further isolation of the antifungal substances.

### 3.2. Molecular Identification of the SFC100166 Isolate

**Figure 1:**
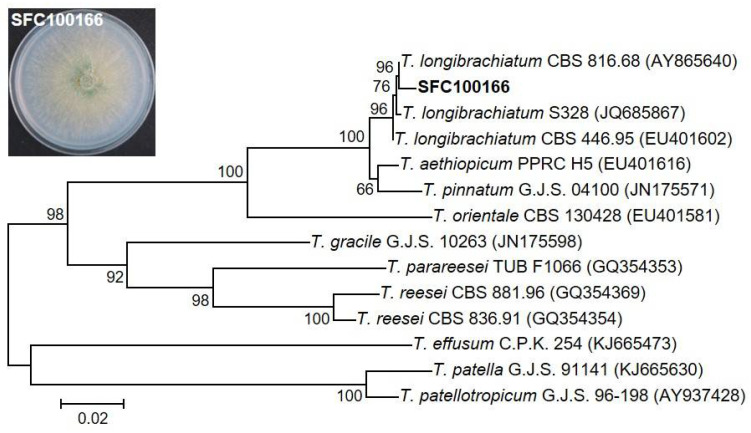
Phylogenetic analysis of the SFC100166 isolate. An inset box showed the SFC100166 isolate culture grown on PDA. Phylogenetic analysis by the neighbor-joining method was performed based on the translation elongation factor 1-α (*TEF1*) gene sequences of SFC100166 and the other 13 *Trichoderma* species. NCBI accession numbers of each sequence are in parentheses. The scale bar indicates the number of nucleotide substitutions per site.

For the molecular identification of the SFC100166 isolate, the *TEF1* gene region was amplified from the gDNA of the SFC100166 isolate, and the resulting sequence (482 bp of the amplicon) was deposited to GenBank (http://www.ncbi.nlm.nih.gov/Genbank) with an accession number of MN307409. Phylogenetic analysis based on *TEF1* sequences showed that the SFC100166 isolate belongs to the genus *Trichoderma* and mostly is related to *T. longibrachiatum* CBS 816.68 (AY865640) with a 99.3% similarity. As such, the SFC100166 isolate was identified as *T. longibrachiatum* (Figure 1).

*T. longibrachiatum* is a soil-borne fungus belonging to the anamorphic genus *Trichoderma* (Hypocreales, Ascomycota). Several strains of *T. longibrachiatum* have been reported as promising biocontrol agents against phytopathogenic fungi, bacteria, and nematodes [15,24,25,26,27,28]. To date, a variety of new antibiotics, including peptides, polyketides, and terpenes, have been isolated from *T. longibrachiatum*, which led to the recognition of the importance of *T. longibrachiatum* as a potential source of novel antibiotics [7,8,15].

### 3.3. Structure Determination of the Active Compounds Isolated from Trichoderma longibrachiatum SFC100166

**Figure 2:**
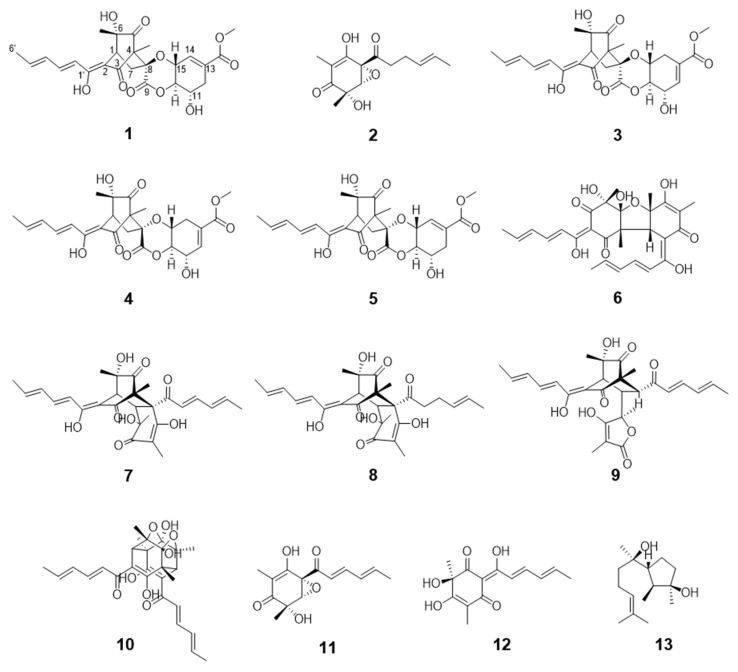
Chemical structures of compounds **1**–**13** isolated from the fungus *Trichoderma longibrachiatum* SFC100166.

The two organic extracts obtained from the culture filtrate of *T. longibrachiatum* SFC100166 were further fractionated and purified by various chromatographic processes following the guidance of in vitro antifungal assays. Consequently, we isolated a total of 13 compounds (Figure 2) and determined the chemical structures of the purified compounds by analyzing the spectroscopic data (NMR, MS, and CD) and by comparing them with previously reported values in the literature. Of the 13 compounds, compound **1** was a newly identified compound, and compound **2** was found for the first time in a natural resource.

**Figure 3:**
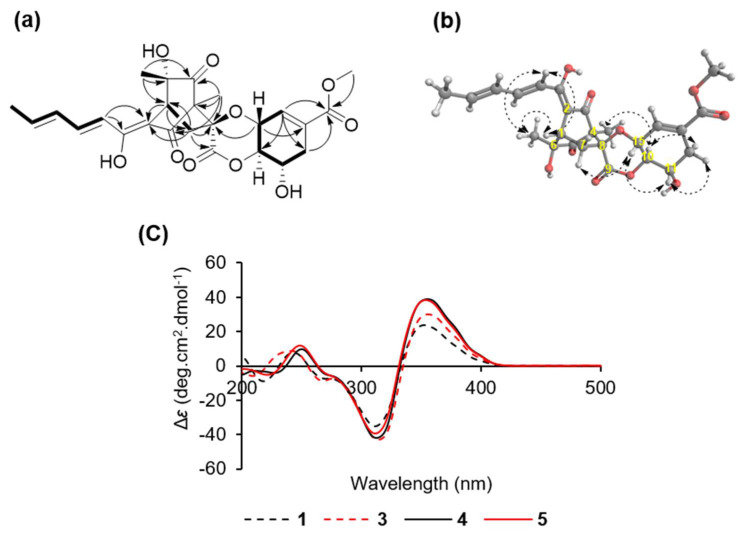
(**a**) Key HMBC (arrow) and COSY (bold line) correlations of compound **1**; (**b**) NOE (dashed arrow) correlations of compound **1**; (**c**) CD spectra of compounds **1** and **3**–**5**.

Compound **1** was obtained as a yellow amorphous powder and has a molecular formula of C_25_H_28_O_10_, which was established by HRESIMS analysis from a molecular ion at *m/z* 511.1580 [M + Na]^+^ (calculated *m/z* 511.1580 for C_25_H_28_O_10_Na) (Figure S2). The details of the ^1^H-NMR, ^13^C-NMR, and HMQC spectra of **1** displayed signals for three methyls (*δ*_H_ 1.23, *δ*_C_ 24.9; *δ*_H_ 1.25, *δ*_C_ 8.3; *δ*_H_ 1.92, *δ*_C_ 18.9), two methylenes (*δ*_H_ 2.24 and 3.13, *δ*_C_ 38.0; *δ*_H_ 2.32 and 2.95, *δ*_C_ 34.4), one methine (*δ*_H_ 3.38, *δ*_C_ 41.4), one methoxyl (*δ*_H_ 3.77, *δ*_C_ 52.7), three oxymethines (*δ*_H_ 4.09, *δ*_C_ 67.1; *δ*_H_ 4.24, *δ*_C_ 84.1; *δ*_H_ 4.66, *δ*_C_ 70.3), and five olefinic methines (*δ*_H_ 6.26, *δ*_C_ 140.6; *δ*_H_ 6.44, *δ*_C_ 132.3; *δ*_H_ 6.46, *δ*_C_ 119.3; *δ*_H_ 6.57, *δ*_C_ 134.2; *δ*_H_ 7.36, *δ*_C_ 143.8), and the remaining 10 quaternary carbons were observed at *δ*_C_ 69.2, 74.6, 85.8, 111.1, 131.7, 167.2, 168.3, 172.2, 195.4, and 206.8 (Table 1 and Figures S3–S5). The structure of **1** was further constructed with ^1^H-^1^H COSY and HMBC experiments (Figures S6 and S7). The HMBC correlations from H-1 to C-2, C-3, C-5, C-6, and C-8 and from H-7 to C-2, C-4, C-8, and C-9, in conjunction with a strong correlation in the ^1^H-^1^H COSY between H-1 and H-7, confirmed that **1** had a bicyclo[2.2.2]octane ring system (Figure 3a). Two methyl groups with proton signals at *δ*_H_ 1.23 and 1.25 were attached to C-6 and C-4 of the bicyclo[2.2.2]octyl moiety, respectively, due to their following correlations in the HMBC spectrum: from 6-CH_3_ to C-1, C-5, and C-6 and from 4-CH_3_ to C-3, C-4, C-5, and C-8. The ^1^H-^1^H COSY cross-peaks between H-6′/H-5′, H-5′/H-4′, H-4′/H-3′, and H-3′/H-2′ and the HMBC correlations from H-2′ to C-1′ and C-2 indicated the presence of an enol-sorbyl chain, and its position was located at C-2. In addition, a methoxyl carbonyl group was easily recognized, and it was connected to a cyclohexene at C-13 based on the correlations from the methoxyl, H-12, and H-14 to the carbonyl at *δ*_C_ 167.2 in the HMBC spectrum and cross-peaks between H-10/H-11, H-11/H-12, H-14/H-15, and H-15/H-10 in the ^1^H-^1^H COSY spectrum.

**Figure 4:**
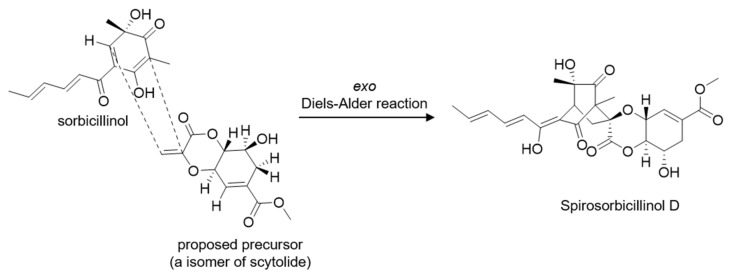
A biosynthetic proposal for compound **1** [31].

A linkage of this fragment and bicyclo[2.2.2]octyl moiety via oxygen atom was determined by a key HMBC correlation from H-15 to C-8. Thus, the planar structure of **1** was established as shown in Figure 2 and Figure 3. It was the same as that of spirosorbicillinol C (**5**), but there are small differences in the chemical shifts of positions C-7, C-8, C10, and C-15 suggesting that the structure of **1** is different from spirosorbicillinol C (**5**) at the stereochemistry of position 8 (Table 1). Indeed, the relative stereochemistry of **1** was confirmed by the analysis of the NOESY spectrum (Figure 3b and Figure S8). The geometries of the double bonds C-2′/C-3′ and C-4′/C-5′ in the enol-sorbyl side chain were determined to be *E*, *E* based on coupling constants *J*_H-2′/H-3′_ = 14.9 Hz and the NOESY correlation between H-3′ and H-5′. The large coupling constants *J*_H-10/H-11_ = 10.4 Hz and *J*_H-10/H-15_ = 8.5 Hz indicated that H-10/H-11 and H-10/H-15 were in an *anti*-orientation. Moreover, the NOESY correlation of H-11 and H-15 suggested that they were oriented in a 1,3-diaxial-like orientation. The relative stereochemistry of C-1/C-4/C-6/C-8/C-10/C-11/C-15 was determined to be 1*S*/4*R*/6*S*/ 8*S*/10*R*/11*S*/15*R* as in the case of the known compound spirosorbicillinol A (**3**) as follows: 6-CH_3_ and C-2 were gauche by the NOESY correlations from 6-CH_3_ to H-2′ and H-1; there were strong HMBC correlations from H-7a to C-6 and from H-7b to C-2, while there was no correlation between H-7a and C-2 and a weak correlation between H-7b and C-6; in addition, a strong HMBC correlation from H-7b to C-9 and no correlation from H-7b to C-9 revealed that H-7a and C-2 were gauche, and H-7b and C-9 were eclipsed; the remaining stereochemistry of the other positions was established by the strong NOESY correlations from H-10 to 4-CH_3_, from H-15 to H-7a, and from H-11 to H-12a and H-10 to H-12b. To elucidate the absolute stereochemistry of **1**, the circular dichroism (CD) spectrum was analyzed and compared with the CD spectra of known sorbicillinoids [29,30]. As shown in Figure 3c, compound **1** and its isomers spirosorbicillinol A–C (**3**–**5**) exhibited a positive cotton effect (CE) at 350–360 nm (Δ*ε* > +24) and a negative CE at 310–320 nm (Δ*ε* < −35), which were in agreement with the 1*R* and 4*S* configurations [29]. The absolute configuration of C-6 was proposed to be the *S*-form as with sorbicillinol, which is a key precursor of sorbicillin-related compounds [31]. Furthermore, compound **1** had an optical rotation value ${\left[\alpha \right]}_{D}^{25}$ = +142 (*c* = 0.05, MeOH), which was closer to that of spirosorbicillinol A (**3**) ${\left[\alpha \right]}_{D}^{25}$ = +184 (*c* = 0.05, MeOH) than that of spirosorbicillinol B (**4**) ${\left[\alpha \right]}_{D}^{25}$ = +428 (*c* = 0.05, MeOH) and spirosorbicillinol C (**5**) ${\left[\alpha \right]}_{D}^{25}$ = +464 (*c* = 0.05, MeOH), suggesting that the position C-8 of compound **1** was the same stereochemistry as spirosorbicillinol A (**3**) (8*S*), whereas spirosorbicillinols B and C have an opposite stereochemistry (8*R*). Based on the analytic results mentioned above, the structure and absolute stereochemistry of the new compound **1** were elucidated as shown in Figure 2 (1*S*, 4*R*, 6*S*, 8*S*, 10*R*, 11*S*, 15*R*), and it was designated as spirosorbicillinol D. Spirosorbicillinol D (**1**) would be biosynthesized by the *exo* intermolecular Diels–Alder reaction from sorbicillinol as a diene and a constitutional isomer of scytolide as a dienophile (Figure 4). Indeed, Kahlert et al. clearly demonstrated that spirosorbicillinols A and B (**3** and **4**) of *Trichoderma reesei* QM6a arise through the *exo* and *endo* Diels–Alder reaction of sorbicillinol with scytolide, respectively [32]. Spirosorbicillinol C (**5**) from the fermentation broth of *Trichoderma* sp. USF-4860 was proposed as a biosynthetic product of the *endo* Diels-Alder reaction between sorbicillinol and the constitutional isomer scytolide [31].

Compound **2** was obtained as a viscous yellow oil with a molecular formula of C_14_H_18_O_5_, which was established by ESIMS analysis from molecular ions at *m/z* 289 [M + Na]^+^ and at *m/z* 265 [M − H]^+^ (Figure S9), which had 2 H more than epoxysorbicillinol (**11**) (C_14_H_16_O_5_). The ^1^H-NMR spectrum of **2** (Figure S10) was closely similar to that of epoxysorbicillinol (**11**) except for the absence of two olefinic double bonds and the presence of two methylenes at *δ*_H_ 2.67 and 2.28, suggesting that the structure of **2** was a reduction product of epoxysorbicillinol (**11**). The ^1^H-NMR spectrum of **2** was compared with a previous report of the synthetic product that is a mediated product provided in the total synthesis of epoxysorbicillinol (**11**). As such, the structure of **2** was assigned as 2′,3′-dihydro-epoxysorbicillinol. In the current study, compound **2** was isolated from a natural resource for the first time [33].

By comparing the spectroscopic data (Table S1) of compounds **3**–**13** with those of the reported values in the literature, 11 compounds were identified as spirosorbicillinol A (**3**) [31], spirosorbicillinol B (**4**) [31], spirosorbicillinol C (**5**) [31], bisvertinolone (**6**) [34,35], bisorbicillinol (**7**) [36], bisvertinoquinol (**8**) [37], trichotetronine (**9**) [30], trichodimerol (**10**) [38], epoxysorbicillinol (**11**) [39], oxosorbicillinol (**12**) [40], and cyclonerodiol (**13**) [41].

### 3.4. Microdilution Broth Assay for the In Vitro Antifungal Activity of the Isolated Compounds (***1**–**13***)

All isolated compounds (**1**–**13**) were evaluated for their in vitro antifungal activity against seven plant pathogenic fungi: *A. brassicicola*, *B. cinerea*, *C. coccodes*, *M. oryzae*, *P. infestans*, *C. cucumerinum*, and *C. destructans*. Of the tested phytopathogenic fungi, the tomato late blight fungus *P. infestans* was the most sensitive to the isolated compounds, except for compounds **9** and **10** which did not have any effects on the growth of the tested fungal pathogens. Compound **6** exhibited the strongest antifungal activity against *P. infestans* with a MIC value of 6.3 μg/mL, followed by compounds **11** and **12** with MIC values of 50 and 25 μg/mL, respectively. Compounds **1**–**5**, **7**, **8**, and **13** showed a moderate antifungal activity against *P. infestans* with MIC values ranging from 100 to 400 μg/mL (Table 3). On the other hand, compound **6** exhibited the strongest antifungal activity against *M. oryzae* with a MIC value of 50 μg/mL, while compounds **7**, **8**, and **11**–**13** showed a moderate antifungal activity against *M. oryzae* with MIC values ranging from 100 to 400 μg/mL (Table 3). Taken together, compounds **6**, **7**, **11**, and **12** showed broad antifungal activities against tested fungal pathogens, and in particular, compound **6** strongly inhibited the growth of the tested microorganisms, with MIC values ranging from 6.3 to 100 μg/mL, except for *B. cinerea* (Table 3).

### 3.5. Antifungal Activity Based on the Compound Structure

The 13 compounds isolated from the fungus *T. longibrachiatum* SFC100166 can be classified into two groups: sorbicillinoids (**1**–**12**) and a terpenoid (**13**). Sorbicillinoids are a family of hexaketide fungal metabolites with a typical or modified sorbyl side chain [29]. Since sorbicillin was isolated as an impurity from *Penicillium notatum* in 1948, which was the first member of the sorbicillinoids group, more than 100 sorbicillinoids have been isolated from marine and terrestrial microbes, such as *Penicillium*, *Trichoderma*, *Aspergillus*, *Acremonium*, *Eurotiomycete*, *Phaeoacremonium*, *Phialocephala*, *Scytalidium*, *Clonostachys*, *Paecilomyces*, and *Verticillium* [29,42,43]. Based on the structural features of various sorbicillinoids, sorbicillinoids can be classified into monomers, dimers, trimers, and hybrids [44,45]. The 12 sorbicillinoids of this study can be classified into three classes: monomeric sorbicillinoids (**2**, **11** and **12**), dimeric sorbicillinoids (**6**–**10**), and hybrid sorbicillinoids (**1** and **3**–**5**). In particular, it has been proposed that bisvertinolone (**6**), showing the strongest antifungal activity, is biosynthesized from oxosorbicillinol (**12**) and sorbicillinol in a Michael-type reaction [46].

It has been reported that many sorbicillinoids show promising biological activities such as anticancer, antimicrobial, antiviral, antitumor, and radical-scavenging activities: bisorbicillinol has shown antioxidant activity, 24-hydroxybisvertinol and bisvertinol have shown anti-inflammatory activity, and ustisorbicillinol B has shown antimicrobial activity [36,44,47,48,49]. In the current study, bisvertinolone (**6**) was the most antifungal agent against the tested phytopathogenic fungi, followed by oxosorbicillinol (**12**) and epoxysorbicillinol (**11**). Similarly, it has been reported that bisvertinolone (**6**) acts as a morphological malformation inducer in growing hyphal cells of *P. capsici* by inhibiting *β*-1,6-glucan biosynthesis [34]. Although it has been reported that oxosorbicillinol (**12**) exhibits a weak antibacterial activity against the Gram-positive bacteria *Staphylococcus aureus* and *Bacillus subtilis* with no effects on fungi and algae [50], oxosorbicillinol (**12**) in this study showed a moderate antifungal activity with MIC values ranging from 25 to 400 μg/mL against phytopathogenic fungi, such as *C. coccodes*, *M. oryzae*, and *P. infestans.*

### 3.6. Disease Control Efficacy of the Isolated Compounds

**Figure 5:**
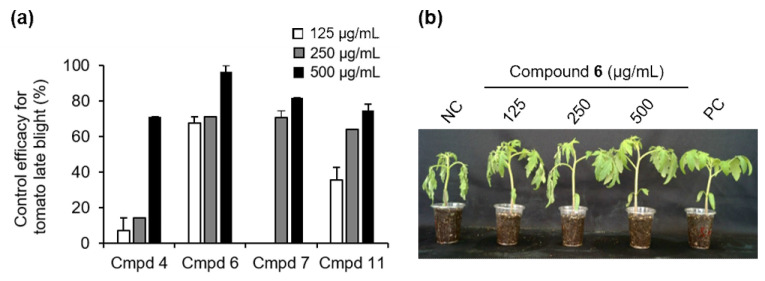
Effects of compounds **4**, **6**, **7**, and **11** on the development of tomato late blight. (**a**) Control efficacy of compounds **4**, **6**, **7**, and **11** against tomato late blight. The bars represent the mean ± standard deviation of two runs with three replicates. (**b**) Representatives of plants treated with compound **6**. Plants were inoculated with a spore suspension of *Phytophthora infestans* 1 day after treatment with compound **6**. NC, treatment with the 0.025% Tween 20 solution containing 5% MeOH was used as a negative control; PC, a chemical fungicide dimethomorph (10 µg/mL) was used as a positive control.

Based on the results of the in vitro antifungal activity and the amount of isolated compounds obtained, we examined the disease control efficacy of compounds **4**, **6**, **7**, and **11** as representative of monomers and dimers of sorbicillinoids against TLB at a concentration of 125, 250, and 500 μg/mL. The treatment with compounds **4**, **6**, **7**, and **11** at a concentration of 500 μg/mL exhibited disease control values of 82%, 96%, 71%, and 75%, respectively, compared to the untreated control (Figure 5). Particularly, compound **6** exhibited a potent disease control value of 68% even at a low concentration of 125 μg/mL (Figure 5). In addition to the disease control effects, no phytotoxic symptoms from the tested compounds were observed on the treated tomato plants. Previously, Meng et al. described that several sorbicilinoids, including bisvertinolone (**6**), identified from rice false smut pathogen *Ustilaginoidea virens* showed a strong inhibitory effect on germ elongation of rice and lettuce seeds [29].

## 4. Conclusions

Herein, we described the isolation and identification of thirteen metabolites from the culture filtrate of *T. longibrachiatum* SFC100166 showing inhibitory effects on the development of RCB, TGM, and TLB. Of the 13 active substances, compound **1** was a new compound and was designated as spirosorbicillinol D, and compound **2** was a new natural compound and was designated as 2′,3′-dihydro-epoxysorbicillinol. In terms of the in vitro and in vivo antifungal activities, compound **6** (bisvertinolone) showed promising antifungal activity in vitro and also reduced the development of TLB disease compared to the untreated control. Taken together, our results revealed that the culture filtrate of *T. longibrachiatum* SFC100166 and its metabolites could be useful sources for the development of new natural agents to control fungal diseases on plants.

## Figures and Tables

**Table 1:** ^1^H NMR data (400 MHz) ^a^ of compound **1** and ^13^C NMR data (100 MHz) of compounds **1** and **3**–**5** in MeOH-*d*_4_.

Position	1		3	4	5
	*δ*_H_, (*J* in Hz)	*δ*_C_	*δ*_C_	*δ*_C_	*δ*_C_
1	3.38, overlapped *	41.4	41.4	41.2	41.2
2		111.1	111.1	111.4	111.2
3		195.4	195.5	196.2	196.1
4		69.2	69.3	70.9	70.9
5		206.8	206.7	207.2	207.2
6		74.6	74.6	74.9	74.9
7	2.24, dd (14.2, 3.7) 3.13, dd (14.2, 2.3)	38.0	37.2	40.4	40.2
8		85.8	84.3	82.6	83.7
9		172.2	172.0	171.0	171.2
10	4.24, dd (10.4, 8.5)	84.1	85.9	84.3	82.9
11	4.09, td (9.7, 6.8)	67.1	70.6	70.7	67.1
12	2.32, m 2.95, dd (18.3, 6.9)	34.4	139.6	139.3	34.0
13		131.7	129.0	128.8	130.8
14	6.57, s	134.2	30.5	31.4	134.2
15	4.66, m	70.3	67.1	71.3	74.9
1′		168.3	168.3	167.9	167.9
2′	6.46, d (14.9)	119.3	119.3	119.5	119.5
3′	7.36, dd (14.9, 10.9)	143.8	143.8	143.2	143.3
4′	6.44, m	132.3	132.3	132.3	132.3
5′	6.26, dt (14.6, 7.0)	140.6	140.6	140.1	140.2
6′	1.92, d (6.8)	18.9	18.9	18.9	18.9
4-CH_3_	1.25, s	8.3	8.1	8.8	8.6
6-CH_3_	1.23, s	24.9	24.8	24.8	24.8
14-COOCH_3_	3.77, s	52.7	52.7	52.7	52.7
14-COOCH_3_		167.2	167.4	167.4	167.3

^a^ Assignment aided by ^1^H-^1^H COSY, HSQC, and HMBC experiments. * overlapped with MeOH-*d*_4_ signal.

**Table 2:** Plant disease control efficacy of the SFC100166 culture filtrate and its organic solvent extracts.

Treatment	Conc. (µg/mL)	Disease Control Value (%) ^a^					
		RCB ^b^	TGM	TLB	WLR	BPM	PAN
Culture filtrate	1-fold dilution	85 ± 2	94 ± 6	90 ± 2	27 ± 3	25 ± 2	15 ± 3
EtOAc extract	1000	88 ± 5	95 ± 2	100	60 ± 4	0	60 ± 2
BuOH extract	1000	25 ± 1	80 ± 1	100	33 ± 1	0	35 ± 5
Water extract	1000	25 ± 1	0	42 ± 2	0	0	0
Blasticidin-S	1	90 ± 2	*–* ^c^	*–*	*–*	*–*	*–*
	50	100	*–*	*–*	*–*	*–*	*–*
Fludioxonil	5	*–*	88 ± 5	*–*	*–*	*–*	*–*
	50	*–*	100	*–*	*–*	*–*	*–*
Dimethomorph	2	*–*	*–*	85 ± 2	*–*	*–*	*–*
	10	*–*	*–*	100	*–*	*–*	*–*
Flusilazole	2	*–*	*–*	*–*	43 ± 9	*–*	*–*
	10	*–*	*–*	*–*	88 ± 2	*–*	*–*
Benomyl	1	*–*	*–*	*–*	*–*	87 ± 5	*–*
	100	*–*	*–*	*–*	*–*	100	*–*
Dithianon	10	*–*	*–*	*–*	*–*	*–*	40 ± 1
	50	*–*	*–*	*–*	*–*	*–*	88 ± 4

^a^ Disease control values (%) represent the mean ± standard deviation of two runs with three replicates. ^b^ RCB, rice blast; TGM, tomato gray mold; TLB, tomato late blight; WLR, wheat leaf rust; BPM, barley powdery mildew; PAN, pepper anthracnose. ^c^ –, not tested.

**Table 3:** In vitro antifungal activity of compounds **1**–**13** against phytopathogenic fungi.

Phytopathogenic Fungi	MIC (µg/mL)													
	1	2	3	4	5	6	7	8	9	10	11	12	13	PC ^a^
*Alternaria brassicicola*	*–* ^b^	*–*	*–*	*–*	*–*	100	400	*–*	*–*	*–*	*–*	400	*–*	6.3
*Botrytis cinerea*	*–*	*–*	*–*	*–*	*–*	*–*	*–*	*–*	*–*	*–*	200	*–*	*–*	50
*Cladosporium cucumerinum*	*–*	*–*	*–*	*–*	*–*	50	*–*	*–*	*–*	*–*	*–*	*–*	*–*	1.6
*Colletotrichum coccodes*	*–*	*–*	*–*	*–*	*–*	12.5	*–*	*–*	*–*	*–*	400	200	*–*	6.3
*Cylindrocarpon destructans*	*–*	*–*	*–*	*–*	*–*	25	*–*	*–*	*–*	*–*	*–*	400	*–*	100
*Magnaporthe oryzae*	*–*	*–*	*–*	*–*	*–*	50	200	200	*–*	*–*	200	100	400	6.3
*Phytophthora infestans*	400	400	400	400	400	6.3	100	200	*–*	*–*	50	25	400	1.6

^a^ PC, blasticidin-S was used as a positive control. ^b^ –, MIC > 400 μg/mL.

## Supplementary Materials

The following are available online at https://www.mdpi.com/article/10.3390/jof7060428/s1, Figure S1: Isolation scheme of compounds **1**–**13** from the culture filtrate of *Trichoderma longibrachiatum* SFC100166, Figures S2–S10: NMR spectral data and MS spectrum of compounds **1** and **2**, Table S1: Spectroscopic data of compounds **3**–**13**.
