# Supplementary material for: In Vitro and In Vivo Antifungal Activity of Sorbicillinoids Produced by Trichoderma longibrachiatum
Source: J Fungi (Basel). 2021 May 28;7(6):428. doi: 10.3390/jof7060428 (PMC8229967; doi:10.3390/jof7060428)
Supplement: Supplementary file 1 [file jof-07-00428-s001.zip › jof-1222338-supplementary.pdf]

In vitro* and *In vivo* Antifungal Activity of Sorbicillinoids Produced by *Trichoderma longibrachiatum

Men Thi Ngo,[1,2] Minh Van Nguyen,[1,2] Jae Woo Han,[1] Myung Soo Park[3], Hun Kim[1,2,]* and Gyung Ja Choi[1,2,]*

[1] *Center for Eco-friendly New Materials, Korea Research Institute of Chemical Technology, Daejeon 34114, Korea*

[2] *Department of Medicinal Chemistry and Pharmacology, University Science and Technology, Daejeon 34113, Korea*

[3] *Department of School of Biological Sciences, Seoul National University, Seoul 08826, Korea*

* Correspondence: hunkim@krict.re.kr and kjchoi@krict.re.kr

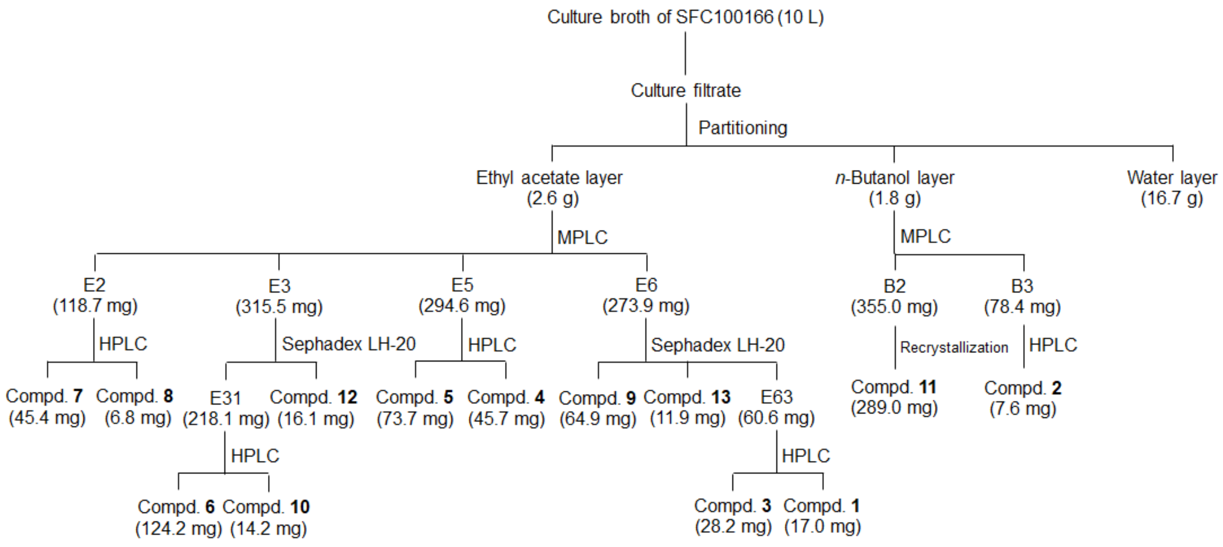

Figure S1. Isolation scheme of compounds **1**–**13** from the culture filtrate of *Trichoderma longibrachiatum* SFC100166.

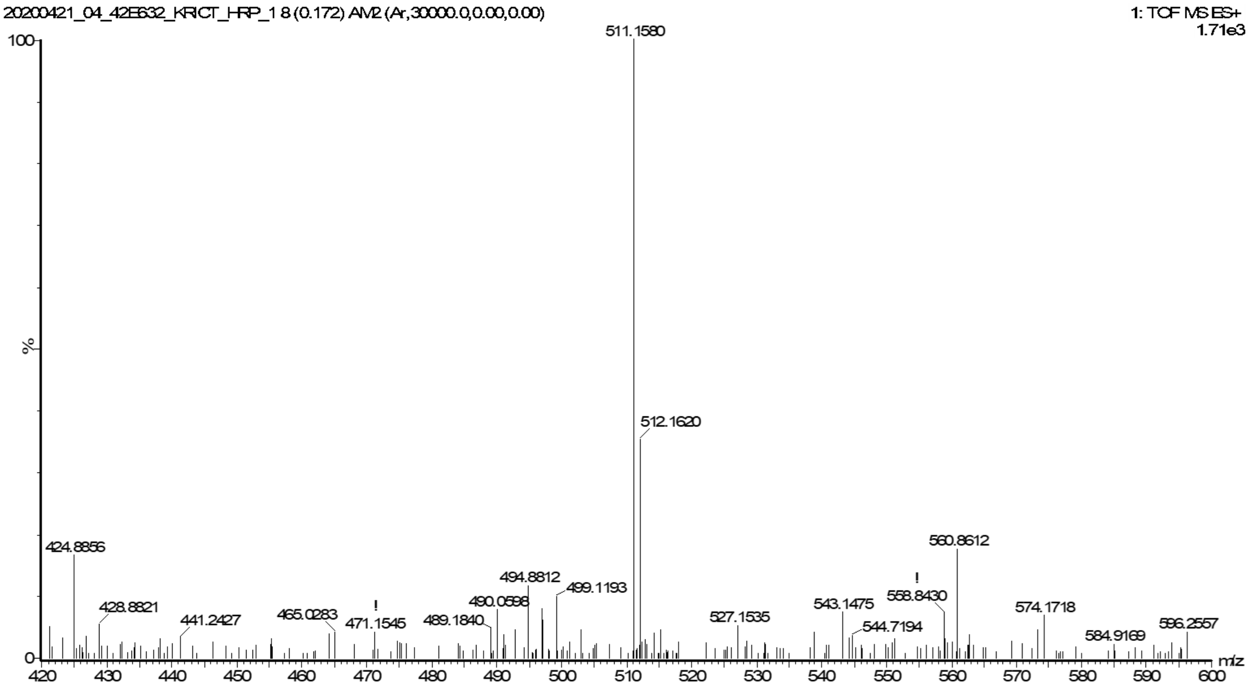

Elemental Composition Report

Single Mass Analysis
Tolerance = 5.0 PPM / DBE: min = -1.5, max = 100.0
Element prediction: Off
Number of isotope peaks used for i-FIT = 3

Monoisotopic Mass, Even Electron Ions
29 formula(e) evaluated with 2 results within limits (all results (up to 1000) for each mass)
Elements Used:
C: 0-30 H: 0-35 O: 0-10 Na: 0-1

Minimum:				-1.5				
Maximum:		100.0	5.0	100.0				
Mass	Calc. Mass	mDa	PPM	DBE	i-FIT	Norm	Conf(%)	Formula
511.1580	511.1580	0.0	0.0	11.5	76.0	0.066	93.64	C25 H28 O10 Na
	511.1604	-2.4	-4.7	14.5	78.7	2.754	6.36	C27 H27 O10

Figure S2. HRESIMS spectrum of compound **1**.

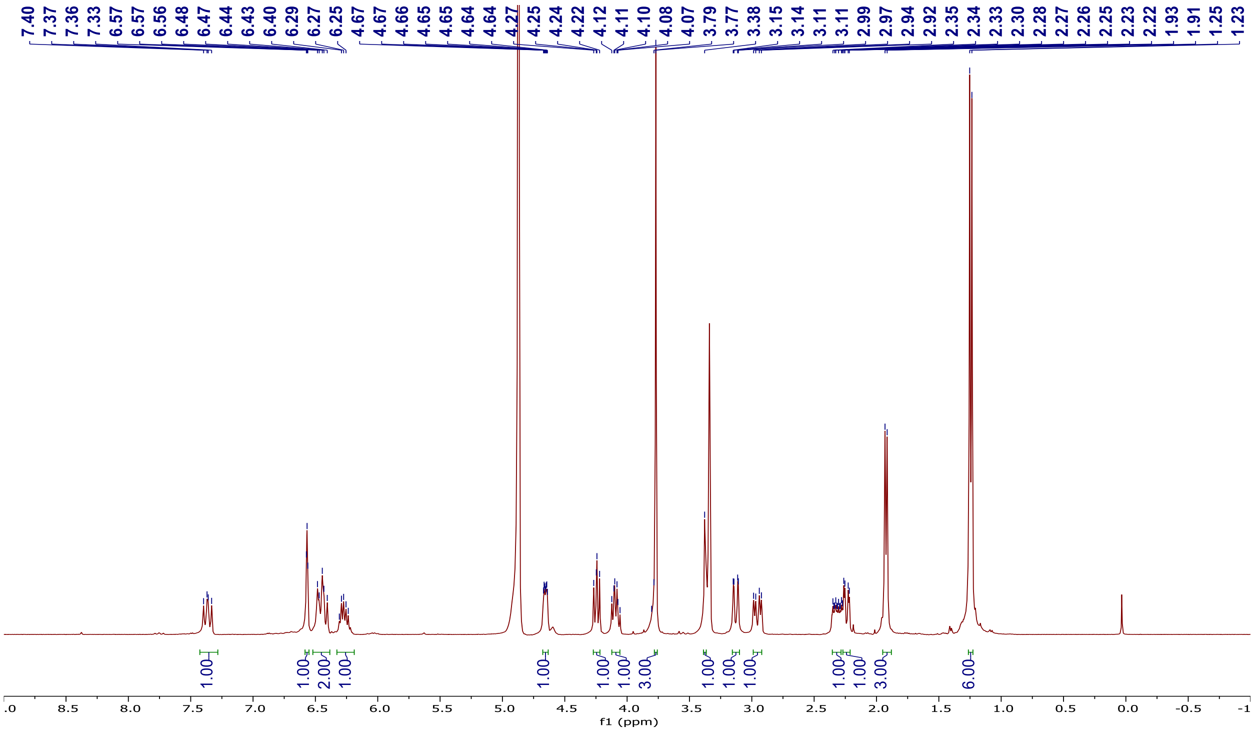

Figure S3. ^{1}H-NMR spectrum of compound **1** (400MHz, methanol-d_4).

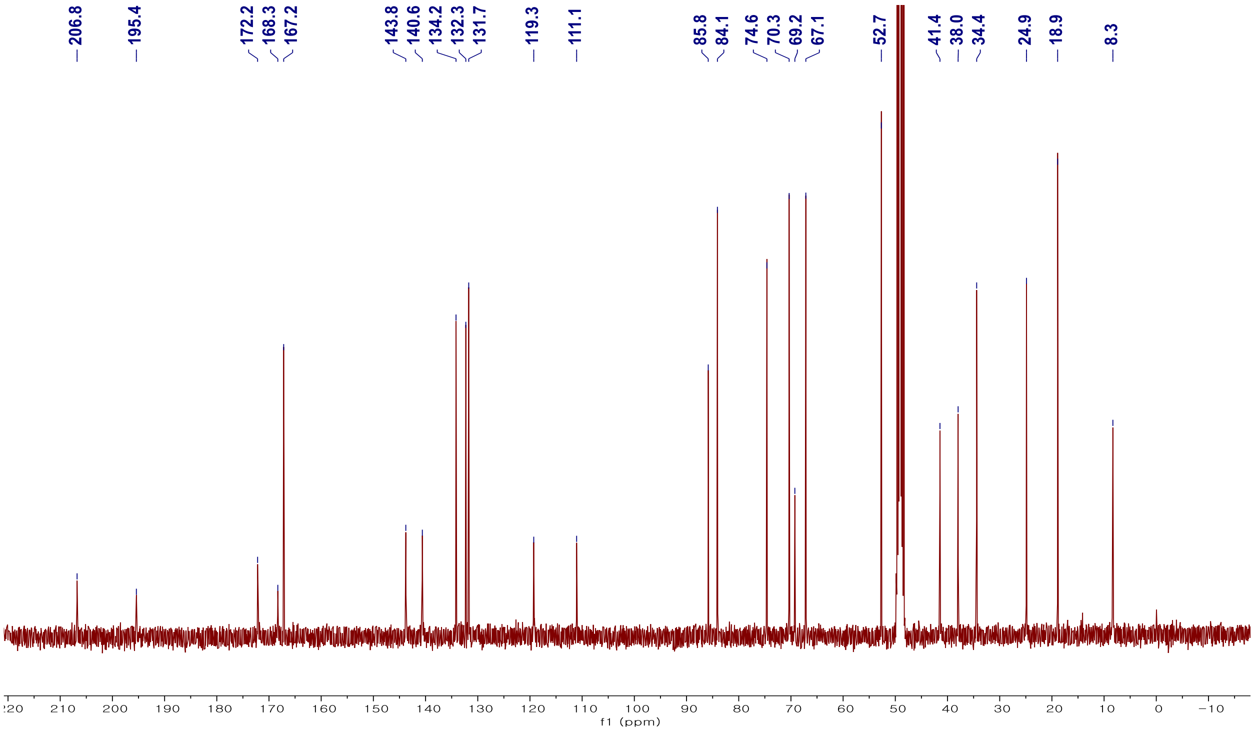

Figure S4. ^{13}C-NMR spectrum of compound **1** (100 MHz, methanol-d_4).

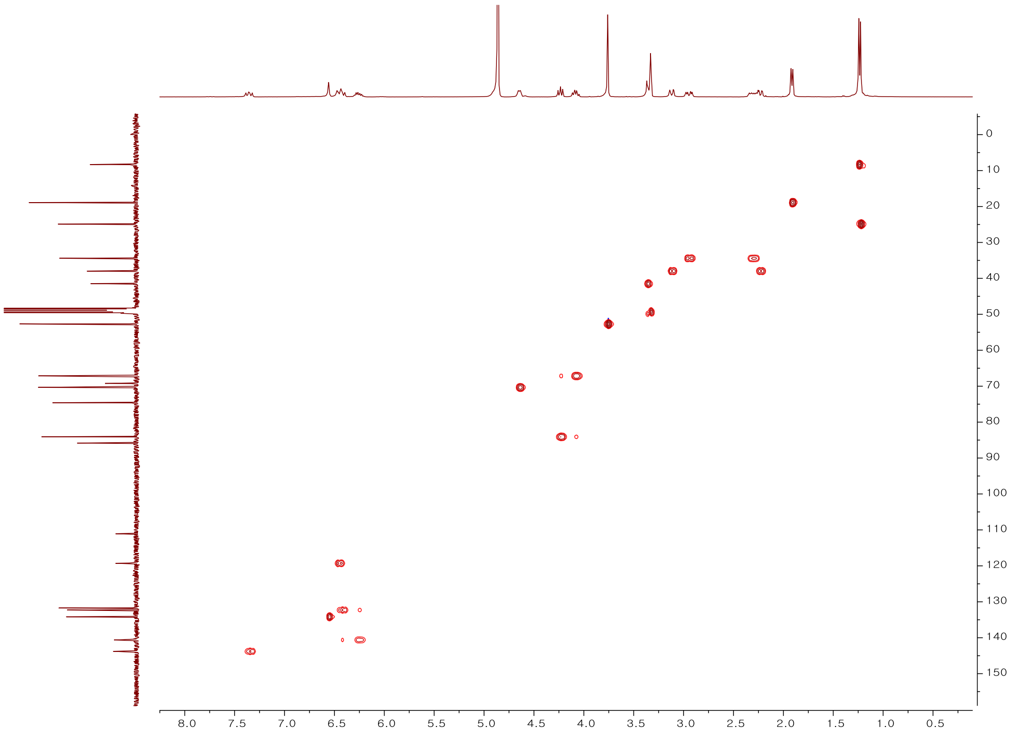

Figure S5. HMQC spectrum of compound **1** (100 MHz, methanol-d_4).

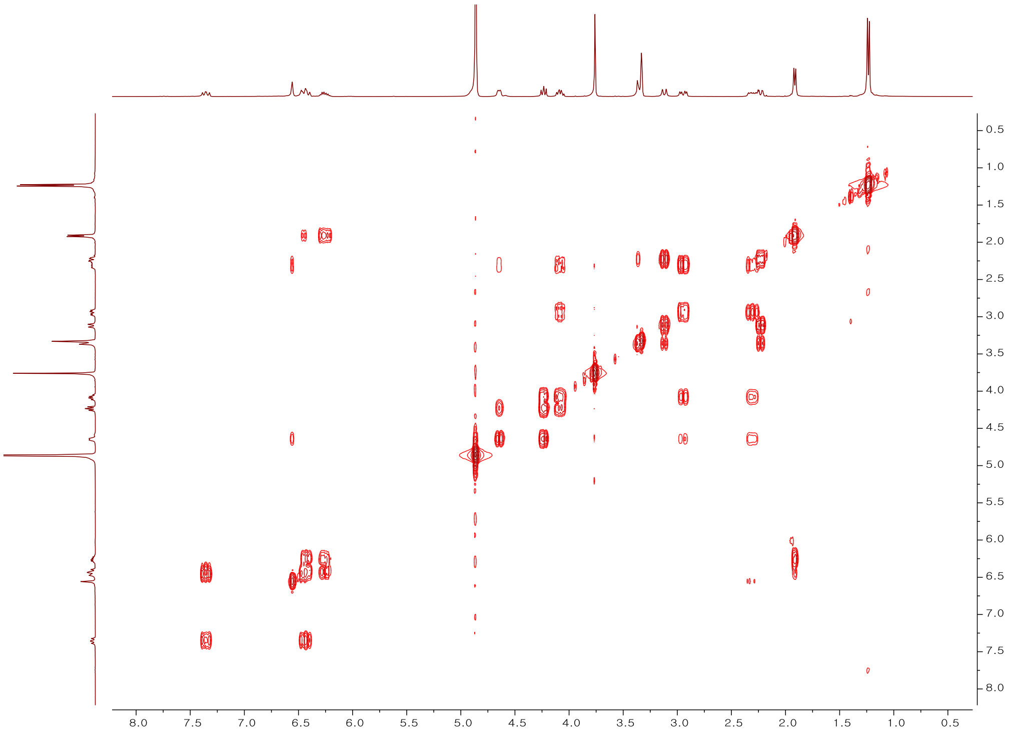

Figure S6. ^{1}H-^{1}H COSY spectrum of compound **1** (400/400MHz, methanol-d_4).

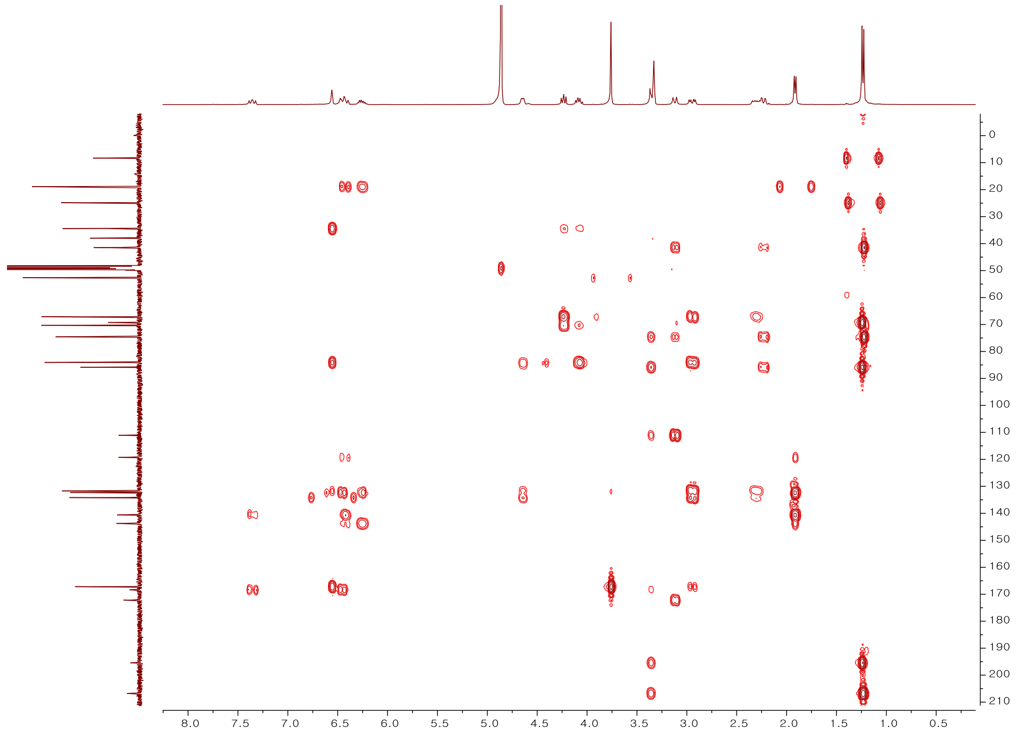

Figure S7. HMBC spectrum of compound **1** (400/100 MHz, methanol-d_4).

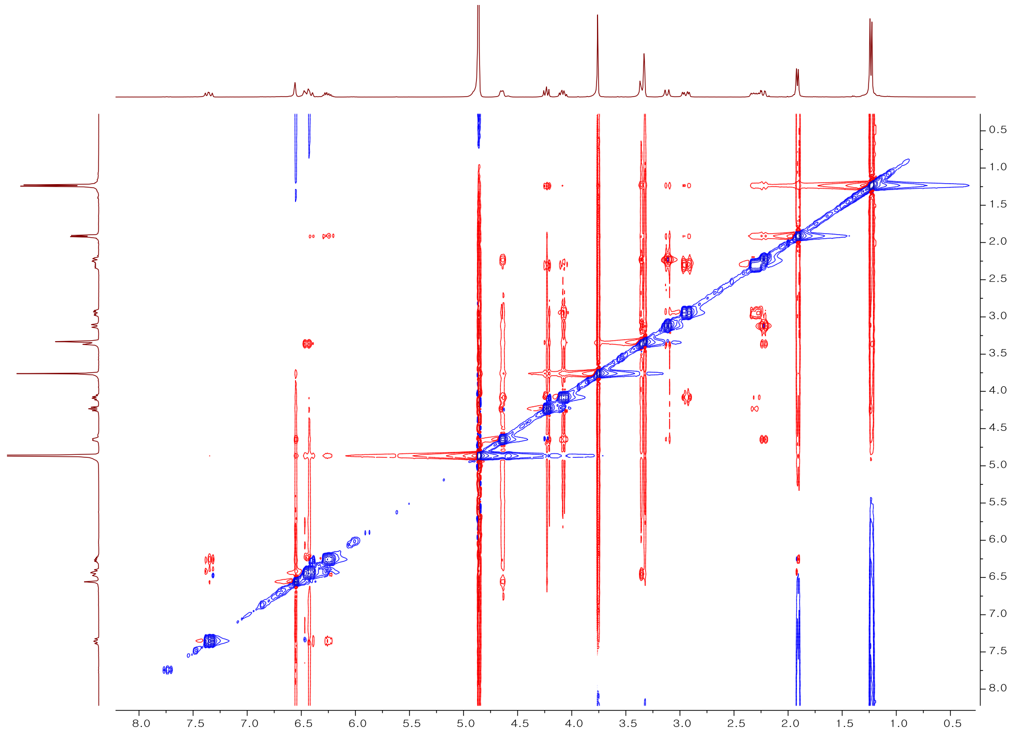

Figure S8. NOESY spectrum of compound **1** (400/100 MHz, methanol-d_4).

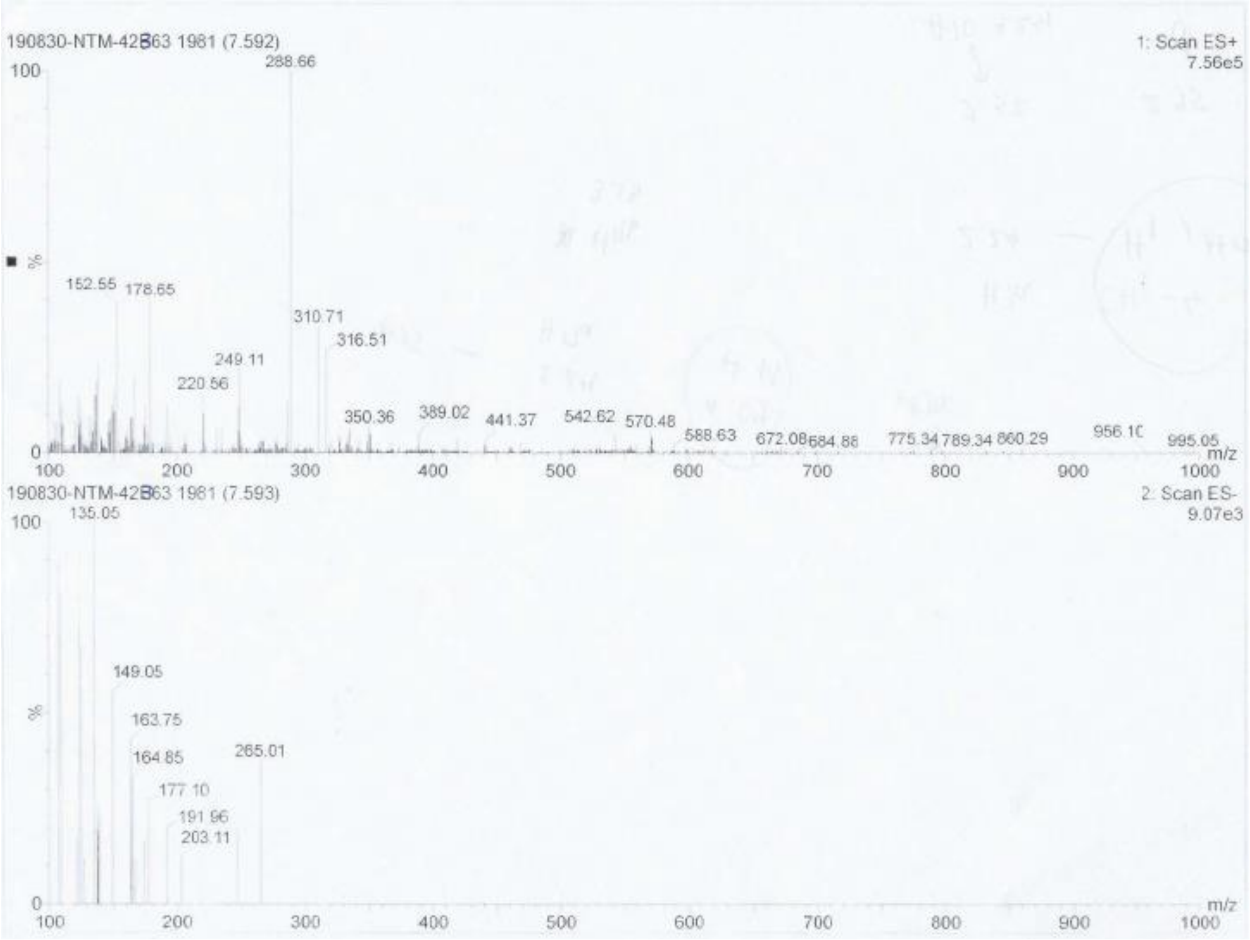

Figure S9. ESIMS spectrum of compound **2**.

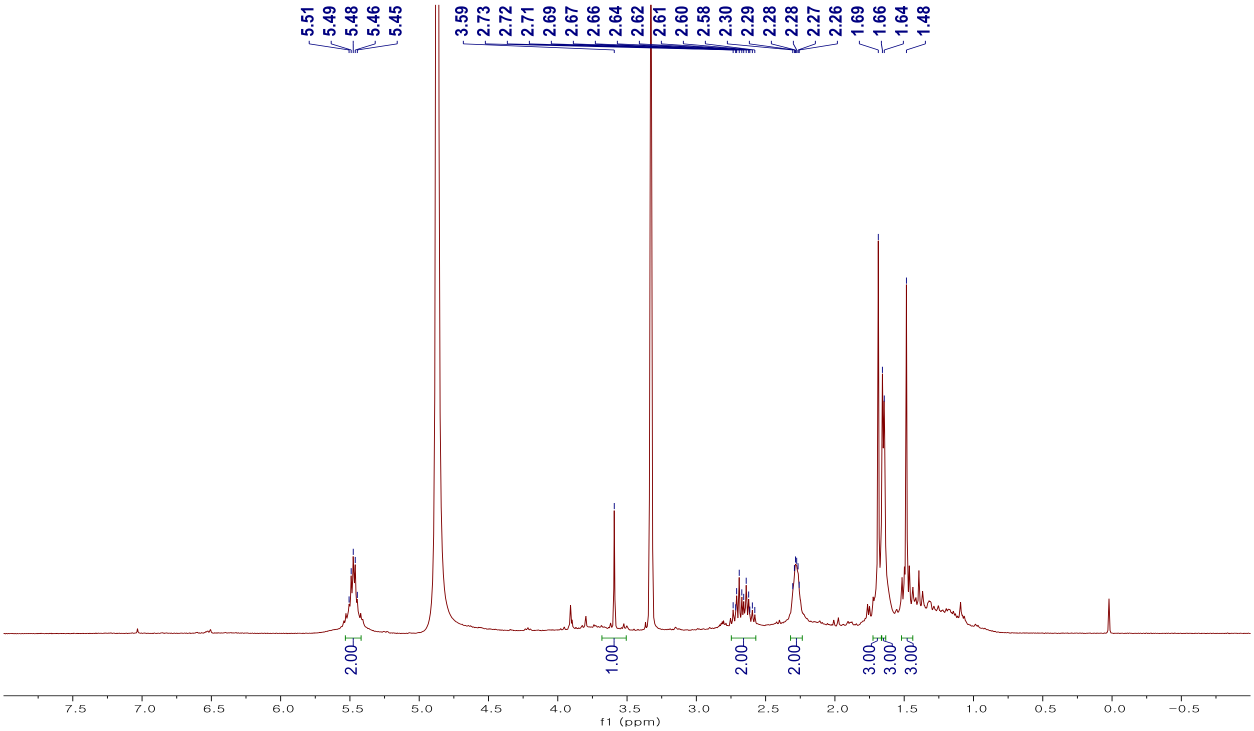

Figure S10. ^{1}H-NMR spectrum of compound **2** (400MHz, methanol-d_4).

Table S1. Spectroscopic data of compounds **3–13**.

Compound	Reference	Description
spirosorbicillinol A (**3**)	[1]	$C_{25}H_{28}O_{10}$ (ESIMS m/z 511 $[M + Na]^+$); ^{13}C-NMR (CD_3OD, 100 MHz) δ_C 206.8, 195.6, 172.0, 168.3, 167.4, 143.8, 140.7, 139.6, 132.3, 129.0, 119.3, 111.1, 85.9, 84.3, 74.6, 70.6, 69.3, 67.1, 52.7, 41.4, 37.2, 30.5, 24.8, 18.9, 8.1.
spirosorbicillinol B (**4**)	[1]	$C_{25}H_{28}O_{10}$ (ESIMS m/z 511 $[M + Na]^+$); ^{13}C-NMR (CD_3OD, 100 MHz) δ_C 207.2, 196.2, 171.0, 167.9, 167.4, 143.2, 140.1, 139.3, 132.3, 128.8, 119.5, 111.4, 84.3, 82.6, 74.9, 71.3, 70.8, 70.7, 52.7, 41.2, 40.4, 31.4, 24.8, 18.9, 8.8.
spirosorbicillinol C (**5**)	[1]	$C_{25}H_{28}O_{10}$ (ESIMS m/z 511 $[M + Na]^+$); ^{13}C-NMR (CD_3OD, 100 MHz) δ_C 207.2, 196.1, 171.2, 167.9, 167.3, 143.3, 140.2, 134.2, 132.3, 130.8, 119.5, 111.2, 83.7, 82.9, 74.9, 74.9, 70.9, 67.1, 52.7, 41.2, 40.2, 34.0, 24.8, 18.9, 8.2.
bisvertinolone (**6**)	[2,3]	$C_{28}H_{32}O_9$ (ESIMS m/z 535 $[M + Na]^+$); ^{13}C-NMR (CD_3OD, 100 MHz) δ_C 201.2, 197.8, 193.1, 186.3, 169.6, 169.1, 148.4, 144.4, 139.9, 137.8, 132.4, 132.4, 123.8, 121.6, 110.7, 109.2, 105.1, 101.9, 80.5, -, 61.3, 55.7, 25.9, 22.9, 19.6, 19.2, 18.8, 7.3.

bisorbicillinol (**7**)	[4]	$C_{28}H_{32}O_8$ (ESIMS m/z 519 $[M + Na]^+$, 495 $[M - H]^-$); ^{1}H-NMR (CD_3OD, 400 MHz) δ_H 7.24 (2H, dt, *J* = 15.0, 11.2 Hz), 6.15-6.50 (6H, m), 3.67 (1H, s), 3.41 (1H, s), 1.90 (6H, m), 1.62 (3H, s), 1.43 (3H, s), 1.21 (3H, s), 1.15 (3H, s).
bisvertinoquinol (**8**)	[5]	$C_{28}H_{34}O_8$ (ESIMS m/z 521 $[M + Na]^+$, 497 $[M - H]^-$); ^{1}H-NMR (CD_3OD, 400 MHz) δ_H 7.23 (1H, m), 6.36 (2H, m), 6.19 (1H, m), 5.46 (2H, m), 3.64 (1H, d, *J* = 2.4 Hz), 3.40 (1H, d, *J* = 2.4 Hz), 2.76 (1H, dt, *J* = 17.9, 7.2 Hz), 2.55 (1H, dt, *J* = 17.9, 7.2 Hz), 2.24 (2H, t, *J* = 6.8 Hz), 1.89 (3H, d, *J* = 6.7 Hz), 1.64 (3H, d, *J* = 6.0 Hz), 1.59 (3H, s), 1.47 (3H, s), 1.20 (3H, s), 1.14 (3H, s).
trichotetronine (**9**)	[6]	$C_{28}H_{32}O_8$ (ESIMS m/z 519 $[M + Na]^+$, 496 $[M - H]^-$); ^{13}C-NMR (CD_3OD, 100 MHz) δ_C 210.1, 202.4, 197.4, 179.5, 176.9, 169.6, 147.8, 145.1, 143.8, 140.8, 132.3, 131.6, 128.4, 119.5, 110.0, 97.5, 84.4, 75.8, 63.5, 52.7, 43.9, 43.4, 24.2, 23.5, 19.1, 18.9, 11.3, 6.5.
trichodimerol (**10**)	[7]	$C_{28}H_{32}O_8$ (ESIMS m/z 519 $[M + Na]^+$, 495 $[M - H]^-$); ^{1}H-NMR (CD_3OD, 400 MHz) δ_H 7.28 (2H, dd, *J* = 14.8, 11.0 Hz), 6.37 (2H, ddd, *J* = 14.8, 11.0, 2.0 Hz), 6.31 (2H, d, *J* = 14.7 Hz), 6.21 (2H, dq, *J* = 14.1, 6.7 Hz), 3.08

		(2H, s), 1.89 (6H, d, *J* = 6.8 Hz), 1.37 (6H, s), 1.35 (6H, s).
epoxysorbicillinol (**11**)	[8]	$C_{14}H_{16}O_5$ (ESIMS m/z 287 $[M + Na]^+$); ^{1}H-NMR (CD_3OD, 400 MHz) δ_H ddd.^{13}C-NMR (CD_3OD, 100 MHz) δ_C 194.4, 188.7, 175.7, 147.3, 144.4, 131.5, 124.4, 107.8, 70.4, 63.7, 62.7, 26.1, 19.0, 7.9.
oxosorbicillinol (**12**)	[9]	$C_{14}H_{16}O_5$ (ESIMS m/z 287 $[M + Na]^+$, 263 $[M - H]^-$); ^{13}C-NMR (CD_3OD, 100 MHz) δ_C 198.6, 192.6, 185.6, 173.8, 145.7, 141.9, 132.4, 124.8, 105.9, 76.5, 28.8, 19.0, 7.3.
cyclonerodiol (**13**)	[10]	^{13}C-NMR (CD_3OD, 100 MHz) δ_C 132.0, 125.9, 82.0, 75.5, 55.4, 45.5, 42.1, 41.4, 26.1, 25.9, 25.1, 24.7, 23.7, 17.7, 15.4.

References

1. Washida, K.; Abe, N.; Sugiyama, Y.; Hirota, A. Novel Secondary Metabolites, Spirosorbicillinols A, B, and C, from A Fungus. *Biosci. Biotechnol. Biochem.* **2009**, 900151–900157.
2. Kontani, M.; Sakagami, Y.; Marumo, S. First *β*-1,6-Glucan Biosynthesis Inhibitor, Bisvertinolone Isolated from Fungus, *Acremonium strictum* and Its Absolute Stereochemistry. *Tetrahedron Lett.* **1994**, *35*, 2577–2580.

3. Trifonov, L.S.; Hilpert, H.; Floersheim, P.; Dreiding, A.S.; Rast, D.M.; Skrivanova, R.; Hoesch, L. Bisvertinols: A New Group of Dimeric Vertinoids from *Verticillium intertextum*. *Tetrahedron.* **1986**, *42*, 3157–3179.
4. Abe, N.; Murata, T.; Hirota, A. Novel DPPH radical scavengers, bisorbicillinol and demethyltrichodimerol, from a fungus. *Biosci. Biotechnol. Biochem.* **1998**, *62*, 661−666.
5. Trifonov, L.S.; Bieri, J.H.; Prewo, R.; Dreiding, A.S.; Hoesch, L.; Rast, D.M. Isolation and Structure Elucidation of Three Metabolites from *Verticillium intertextum*: Sorbicillin, Dihydrosorbicillin and Bisvertinoquinol. *Tetrahedron.* **1983**, *39*, 4243–4256.
6. Shirota, O.; Pathak, V.; Hossain, C.F.; Sekita, S.; Takatori, K.; Satake, M. Structural Elucidation of Trichotetronines: Polyketides Possessing A Bicyclo [2.2. 2] Octane Skeleton with A Tetronic Acid Moiety Isolated from *Trichoderma* sp. *J. Chem. Soc. Perkin Trans. I* **1997**, 2961–2964.
7. Andrade, R.; Ayer, W.A.; Mebe, P.P. The Metabolites of *Trichoderma longibrachiatum*. Part 1. Isolation of the Metabolites and the Structure of Trichodimerol. *Can. J. Chem.* **1992**, *70*, 2526–2535.
8. Sperry, S.; Samuels, G.J.; Crews, P. Vertinoid Polyketides from the Saltwater Culture of the Fungus *Trichoderma longibrachiatum* Separated from A *Haliclona* Marine Sponge. *J. Org. Chem.* **1998**, *63*, 10011–10014.
9. Abe, N.; Yamamoto, K.; Hirota, A. Novel Fungal Metabolites, Demethylsorbicillin and Oxosorbicillinol, Isolated from *Trichoderma* sp. USF-2690. *Biosci. Biotechnol. Biochem.* **2000**, *64*, 620–622.
10. Nozoe, S.; Goi, M.; Morisaki, N. Structure of Cyclonerodiol. *Tetrahedron Lett.* **1970**, *11*, 1293–1296.
